# Smaller particular matter, larger risk of female lung cancer incidence? Evidence from 436 Chinese counties

**DOI:** 10.1186/s12889-022-12622-1

**Published:** 2022-02-18

**Authors:** Huagui Guo, Xin Li, Jing Wei, Weifeng Li, Jiansheng Wu, Yanji Zhang

**Affiliations:** 1 School of Architecture and Urban-rural Planning, Fuzhou University, Fuzhou, 350108 China; 2 Department of Architecture and Civil Engineering, City University of Hong Kong, Hong Kong, China; 3 Earth System Science Interdisciplinary Center, Department of Atmospheric and Oceanic Science, University of Maryland, College Park, MD USA; 4 Department of Urban Planning and Design, The University of Hong Kong, Hong Kong, China; 5 Guangdong - Hong Kong - Macau Joint Laboratory for Smart Cities, Shenzhen, 518000 China; 6 Key Laboratory for Urban Habitat Environmental Science and Technology, Shenzhen Graduate School, Peking University, Shenzhen, 518055 China; 7 Key Laboratory for Earth Surface Processes, Ministry of Education, College of Urban and Environmental Sciences, Peking University, Beijing, 100871 China; 8 School of Humanities and Social Sciences, Fuzhou University, Fuzhou, 350108 China

**Keywords:** PM_1_, PM_2.5_, PM_10_, Lung cancer, China

## Abstract

**Background:**

Many studies have reported the effects of PM_2.5_ and PM_10_ on human health, however, it remains unclear whether particular matter with finer particle size has a greater effect.

**Objectives:**

This work aims to examine the varying associations of the incidence rate of female lung cancer with PM_1_, PM_2.5_ and PM_10_ in 436 Chinese cancer registries between 2014 and 2016.

**Methods:**

The effects of PM_1_, PM_2.5_ and PM_10_ were estimated through three regression models, respectively. Mode l only included particular matter, while Model 2 and Model 3 further controlled for time and location factors, and socioeconomic covariates, respectively. Moreover, two sensitivity analyses were performed to investigate the robustness of three particular matte effects. Then, we examined the modifying role of urban-rural division on the effects of PM_1_, PM_2.5_ and PM_10_, respectively.

**Results:**

The change in the incidence rate of female lung cancer relative to its mean was 5.98% (95% CI: 3.40, 8.56%) for PM_1_, which was larger than the values of PM_2.5_ and PM_10_ at 3.75% (95% CI: 2.33, 5.17%) and 1.57% (95% CI: 0.73, 2.41%), respectively. The effects of three particular matters were not sensitive in the two sensitivity analyses. Moreover, urban-rural division positively modified the associations of the incidence rate of female lung cancer with PM_1_, PM_2.5_ and PM_10_.

**Conclusions:**

The effect on the incidence rate of female lung cancer was greater for PM_1_, followed by PM_2.5_ and PM_10_. There were positive modifying roles of urban-rural division on the effects of three particular matters. The finding supports the argument that finer particular matters are more harmful to human health, and also highlights the great significance to develop guidelines for PM_1_ control and prevention in Chinese setting.

**Supplementary Information:**

The online version contains supplementary material available at 10.1186/s12889-022-12622-1.

## Introduction

Great health concern has been placed on the severe air pollution in China. Particular matters as the dominant air pollutants in Chinese cities (e.g. PM_1_, PM_2.5_ and PM_10_), have already been recognized as the Group I carcinogenic factor to lung cancer diseases in the world [1]. As reported by the State of Global Air 2020, particular matter air pollution has led to the mortality of around 500,000 infants across the world [2]. Despite considerable efforts on the estimates of particular matter effects, especially for PM_2.5_ and PM_10_ [3–5], however, whether finer particular matter has the greater effect on human health has not been well understood in China and across the world.

Several potential mechanisms have been proposed to explain the varying effects of size-fractioned particular matters. Biologically, particular matters including PM_1_, PM_2.5_ and PM_10_, can exert adverse effects on the physical health of human beings by the way of aggregating genetic damage [1]. With regards to the difference in health effects, firstly, there is high ratio of surface area to volume in finer than in coarser particular matters. This enables finer particular matter to more easily approach the deeper places in lung, such as lung alveoli [6, 7]. Secondly, the proportion of toxic chemical composition is usually higher in finer than in coarser particular matters. Such physicochemical property makes finer particular matter more easily cause detrimental effects on lung function and epigenetic alteration [8, 9].

Empirically, few attempts have examined the effects of PMs with different particle sizes. In general, the argument that smaller particular matters have greater effects on human health, is still debated. Many studies tend to support this argument, especially for research investigating the effects of PM_1_, PM_2.5_ and PM_10_ [10–12]. Particularly, a time-series study performed in 65 Chinese cities between 2014 and 2017 indicated that the association with cardiovascular disease was stronger for PM_1_ than for PM_2.5_ and PM_10_ [7]. Similarly, as reported in the 33 Communities Chinese Health Study, the odds ratio of cardiovascular disease associated with a 10 μg/m^3^ increase in PMs was 1.12 (95% CI:1.05, 1.20) for PM_1_, which was higher than 1.06 (95% CI:1.01, 1.11) of PM_2.5_ [13]. By contrast, some studies report the greater effects of particular matters with larger particle sizes [14, 15] or the insignificant effects of some size-fractioned particular matters [16, 17]. For example, a case–crossover study performed in Barcelona of Spain suggested that the effect on cardiovascular mortality during Non-Saharan dust days was smaller for PM_1_ than for PM_2.5_ and PM_10_ [18].

Apart from inconsistent findings above, more efforts are required due to the three reasons. Firstly, of research investigating the varying effects of particular matters with different particle sizes, most are single- or several-site studies [12, 19–21], while nationwide studies are quite limited [7, 22]. Hence, findings from previous studies are still not sufficient to conclude the greater effects of finer particular matters than those of coarser particular matters. Secondly, few studies pay attention to PM_1_ which is the dominant component of severe PM_2.5_ air pollution in Chinese cities [23]. This is partly resulted from the unavailable data on PM_1_, especially at the national scale [7, 10]. Thirdly, it remains unknown whether smaller particular matter has the larger effect on lung cancer which has become the second-order of cancer incidences for the female in China [24], although numerous studies have suggested the effects of PMs (especially for PM_2.5_ and PM_10_) on lung cancer diseases [3, 25–27].

To fill the aforementioned gaps, this work used data collected from 436 Chinse counties between 2014 and 2016 to examine whether finer particular matter has the greater effect on the incidence rate of female lung cancer in China where particular matter air pollution is much more severe than developed countries. To answer the research question, three regression models were developed with different controls of time, location and socioeconomic covariates. We further investigated whether the findings are sensitive to the controls of smoking and drinking behaviors as well as additional air pollutant. Moreover, we tried to answer whether urban-rural division modifies the association of the incidence rate of female lung cancer with each of three particular matters (i.e. PM_1_, PM_2.5_ and PM_10_).

## Data and methods

### Research area

**Fig. 1:**
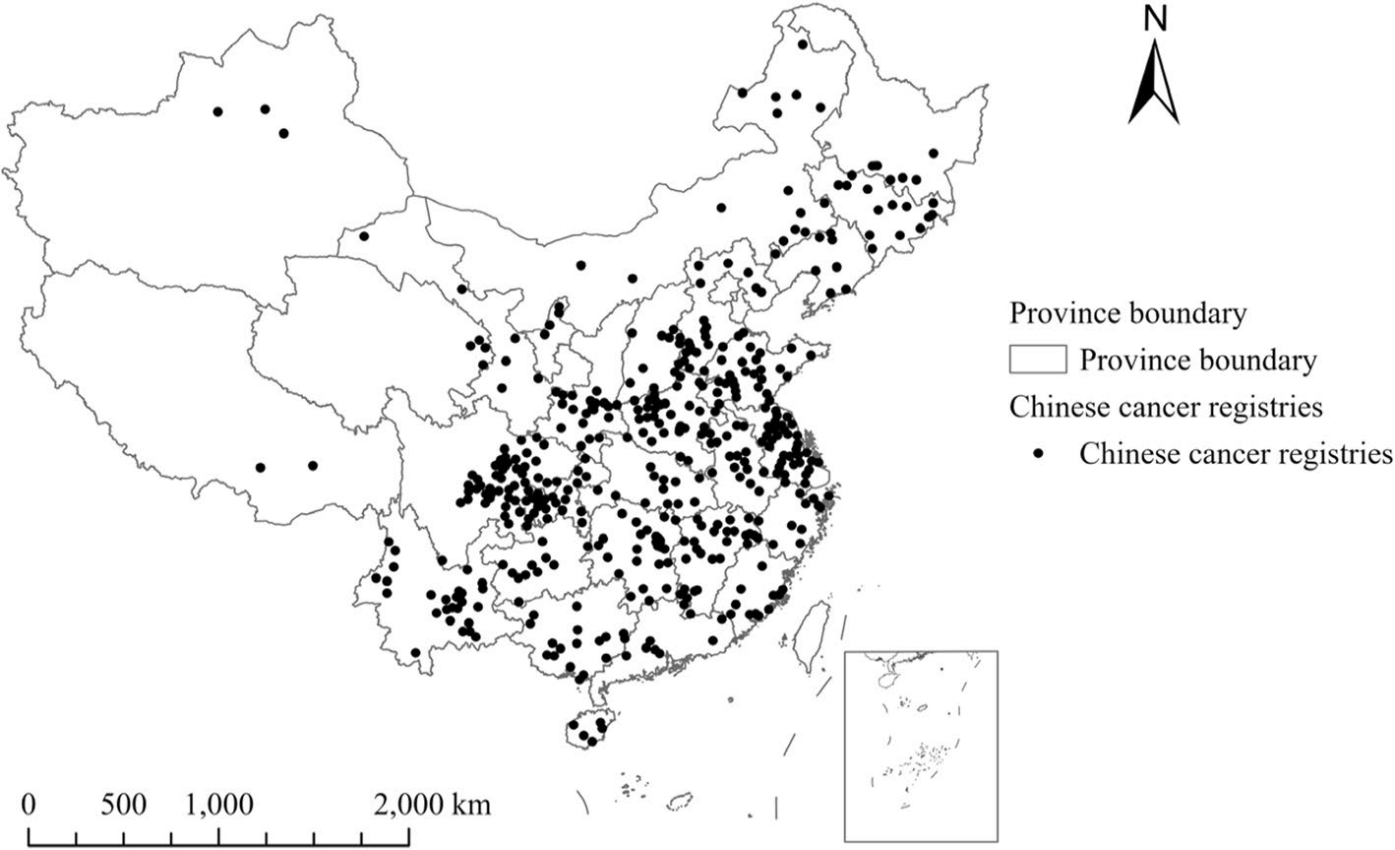
Spatial distributions of 436 Chinese cancer registries between 2014 and 2016

This work focuses on the examination of PM_1_, PM_2.5_ and PM_10_ effects in 436 Chinese cancer registries (Fig. 1). These registries, as shown in Fig. 1, are located in 31 of 34 province-level administrative regions all over China. They are home to around 272.12 million inhabitants in 2016. Among the registries, the number of urban and rural registries (counties and districts, respectively) are 110 and 326, respectively. The selection of 436 registries between 2014 and 2016 is mainly due to the available data on PM_1_, PM_2.5_ and PM_10_ (mainly during 2014–2020), the incidence rate of female lung cancer (2006–2016) and socioeconomic factors (2006–2016). The mean values of PM_1_, PM_2.5_ and PM_10_ concentrations for the selected registries (counties/districts) in 2016 were 34.67 μg/m^3^, 45.80 μg/m^3^ and 90.26 μg/m^3^, respectively.

### Data

#### Ambient PM_1_, PM_2.5_ and PM_10_ concentrations

**Fig. 2:**
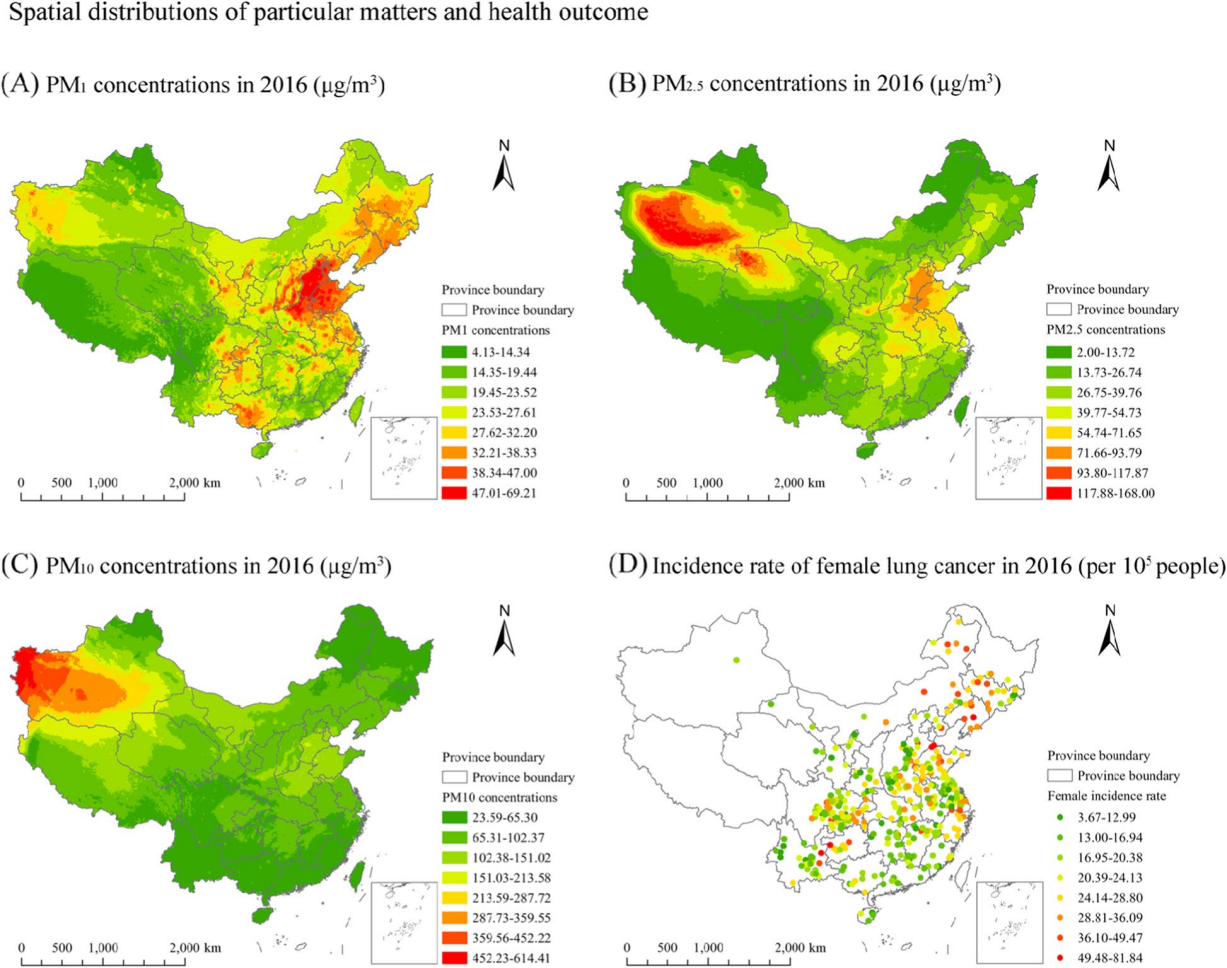
The spatial distributions of PM_1_, PM_2.5_, PM_10_ concentrations as well as the incidence rate of female lung cancer in 2016

Data on PM_1_ concentrations aggregated in the 436 Chinese cancer registries, were obtained from the ChinaHighPM_1_ dataset (that is, the high-resolution and high-quality PM_1_ dataset in China, https://weijing-rs.github.io/product.html). More details of the estimate of PM_1_ concentrations have been well documented [28]. Briefly, a space-time extremely randomized trees model was developed to produce the daily time-series dataset of PM_1_ concentrations at 1 km × 1 km grids from 2014 to 2018 all over China (not include Taiwan, Hong Kong and Macau). During the production of the ChinaHighPM_1_ dataset, data as model input mainly included satellite remote sensing (MAIAC AOD), MEIC pollution emissions, meteorological characteristics, land use and urban form (land use, road, population), and the spatiotemporal terms. Notably, the spatiotemporal terms are used to capture the spatiotemporal autocorrelations of PM_1_ concentrations. As reported from the results of ten-fold cross-validation [28], high consistency exists between the estimated PM_1_ concentrations and PM_1_ measures at daily scale (the coefficient of determination (R^2^ hereafter) = 0.77, root-mean-square error (RMSE hereafter) = 14.6 μg/m^3^). Such high consistency was also observed for the seasonal and annual estimate of PM_1_ concentrations (R^2^ = 0.97, RMSE = 4.1 μg/m^3^). To date, the ChinaHighPM_1_ dataset has been increasingly utilized to estimate PM_1_ effects on the physical health of human beings in China [29–31]. The spatial distributions of PM_1_ concentrations across Mainland China in 2016 are shown in Fig. 2 (A).

We collected data of PM_2.5_ concentrations aggregated in each registry between 2014 and 2016 from the dataset of China Regional Estimates (V4.CH.02). This dataset was public and released by the Atmospheric Composition Analysis Group of Dalhousie University (http://fizz.phys.dal.ca/~atmos/martin/?page_id=140). Details of how the dataset is produced has been well recorded [32]. In short, firstly, AOD was retrieved on the basis of the three satellite instruments. They are the NASA Moderate Resolution Imaging Spectroradiomete, Multi-angle Imaging Spectroradiometer as well as the Sea-Viewing Wide Field-of-View Sensor. Then, the AOD retrieved from the satellite instruments was connected to near-surface PM_2.5_ concentrations through the GEOS-Chem chemical transport model. Hence, the long-term annual time-series dataset of surface PM_2.5_ concentrations at 1 km^2^ spatial resolution is produced. As validated [33], the estimated PM_2.5_ concentrations were highly associated with ground monitoring measurements (R^2^ = 0.81). To date, the dataset of China Regional Estimates (V4.CH.02) has been widely used in PM_2.5_-associated studies in China, including the estimate of health effect as well as the identification of PM_2.5_ driving factors [34, 35]. Figure 2(B) presents the spatial distributions of PM_2.5_ concentrations all over China in 2016.

Annual PM_10_ data for the 436 Chinese cancer registries between 2014 and 2016 were acquired from the ChinaHighPM_10_ dataset (https://weijing-rs.github.io/product.html). The production of PM_10_ dataset, already been well documented [36], is similar to that of PM1. Briefly, a tree-based ensemble learning model was designed to estimate PM_10_ concentrations across China, which combined the MAIAC, factors accounting for the spatiotemporal autocorrelations of PM_10_ as well as the auxiliary data (e.g. meteorological factors, land cover and pollutant emissions). Hence, a time-series and full-coverage dataset of PM_10_ at 1 km × 1 km cell grids from 2013 to 2020 was generated. On the basis of the result of out-of-sample cross validation [36], there is high agreement between PM_10_ estimates and ground-level measurements, with R^2^ and RMSE equal to 0.86 and 24.28 μg/m^3^, respectively. The two values of model performance for out-of-station cross validation were 0.82 and 27.07 μg/m^3^, respectively [36]. To date, there has been the increasing use of the ChinaHighPM_10_ dataset to examine PM_10_’s associations with human health in China [37–39]. The spatial distributions of PM_10_ concentrations all over China in 2016 are exhibited in Fig. 2 (C).

#### Incidence rate of lung cancer for females

We extracted data of health outcome, namely the annual age-standardized incidence rate of trachea, bronchus and lung cancer for females (i.e. the incidence rate of female lung cancer hereafter), from the 2017–2019 China Cancer Registry Annual Reports [24, 40, 41]. To date, the situation of lung cancer has been increasingly severe for the female in China, and such cancer has been the second-order cause of cancer incidence (morbidity) for the female in this country, with the morbidity rate of 42.28 per 100,000 people in 2016 [24]. Meanwhile, most of Chinese studies having connected air pollution to lung cancer diseases focus on the male, while relatively little attention has been placed on the female. Hence, the incidence rate of female lung cancer was selected as the health outcome in this work.

According to the report, health outcome of the present study (the incidence rate of female lung cancer) is defined as the incidence (morbidity) number of lung cancer for the female per 100,000 people per year in a county/district, and then age-standardized on the basis of the Segi’s world population. These annual reports with timely and representative information on cause-specific cancer diseases across China (except Taiwan, Macau and Hong Kong), were public and released by the Chinese Cancer Registry at the National Cancer Centre of China. Particularly, the 2019 China Cancer Registry Annual Report released data of cause-specific cancer diseases for 682 Chinese cancer registries which are dispersed over 31 of 34 Chinese province-level administrative regions [24]. Figure 2 (D) shows the spatial distributions of health outcome (i.e. the incidence rate of female lung cancer) in 2016.

#### Socioeconomic characteristics and smoking and drinking factors

**Fig. 3:**
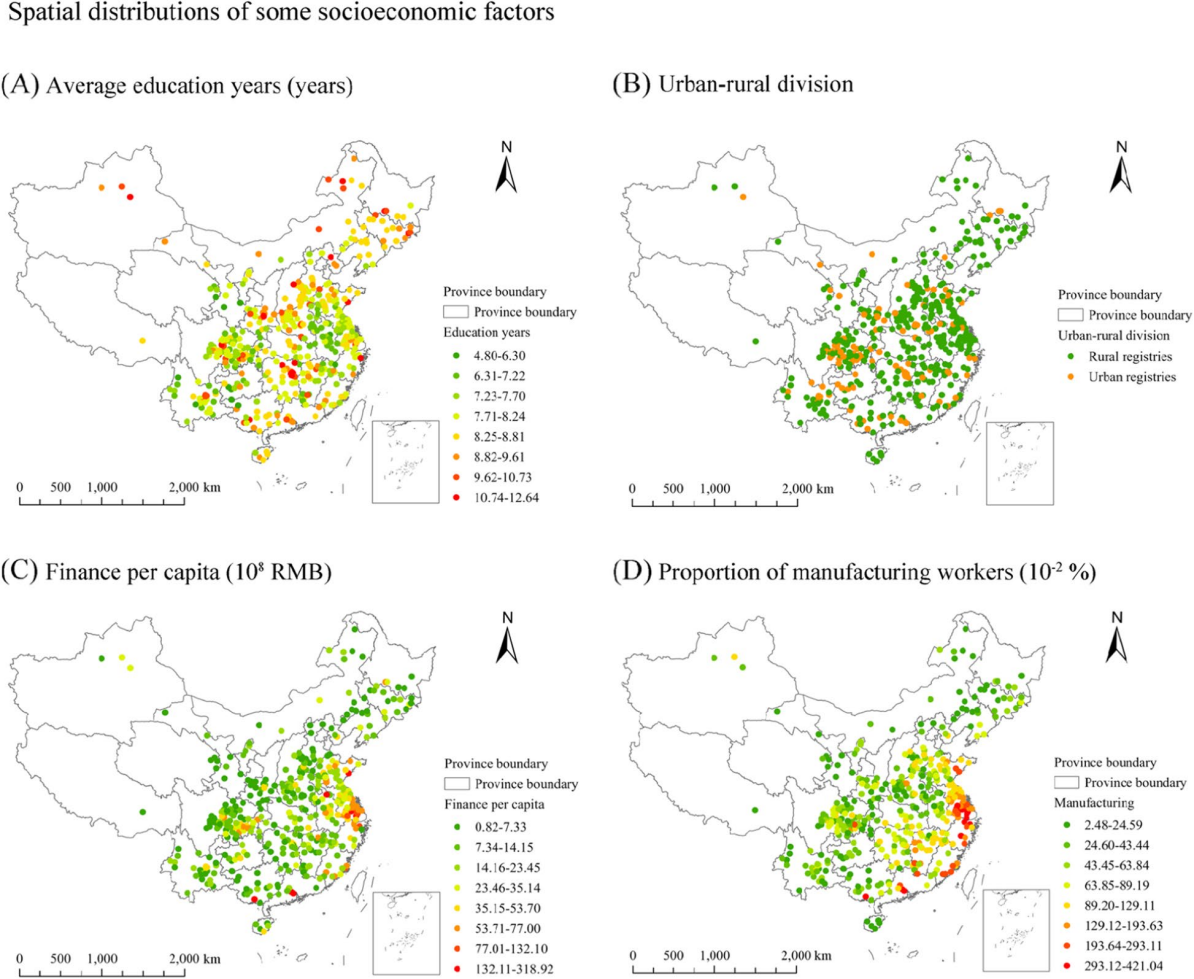
The spatial distributions of educational attainment, urban-rural division, financial level and proportion of manufacturing workers

On the basis of data availability and their reported effects on lung cancer diseases [42, 43], six socioeconomic variables were selected. They are finance per capita (10^9^ RMB), proportions of construction and manufacturing workers (10^− 1^%), population (10^5^ people), average education years (10 years), and urban-rural division (rural group as the reference). These data were extracted from the 2015–2017 China Statistical Yearbooks (County-Level) and the tabulation of the 2010 population census of the People’s Republic of China. The spatial distributions of educational attainment, financial level, urban-rural attributes and proportion of manufacturing workers are presented in Fig. 3.

We extracted smoking and drinking data from the 2015 China Health and Retirement Longitudinal Study (CHARLS) wave3. The CHARLS dataset is public and released by the National School of Development, Peking University (http://charls.pku.edu.cn/en/page/data/2015-charls-wave4), with the aim of providing timely and representative data on health conditions for Chinese people (with the age of 45 and above) at the national scale [44]. According to the CHARLS, the recruited households and individuals reached to 10,257 and 17,708, respectively, and are located in 28 Chinese province-level administrative regions [44]. To date, this national and representative survey has been increasingly employed for the identification of the determinants of human health [45, 46].

### Statistical analysis

Three models were developed to estimate each of the effects of PM_1_, PM_2.5_ and PM_10_ on the incidence rate of female lung cancer. In Model 1 (i.e. univariate model), only PM_1_ (PM_2.5_ or PM_10_) was included. This is to examine whether there is a significant association between PM_1_ (PM_2.5_ or PM_10_) and the incidence rate of female lung cancer. In Model 2, time and location factors were controlled for. This is to test whether the effect of PM_1_ (PM_2.5_ or PM_10_) is still significant after the adjustment of the two factors. In Model 3, we further adjusted for socioeconomic covariates including finance per capita, urban-rural division (as a dummy variable with rural group as the reference), average education years, population as well as proportions of construction and manufacturing workers. This is to examine whether the effect of PM_1_ (PM_2.5_ or PM_10_) is robust to the control of socioeconomic covariates. These factors are chosen mainly according to their associations with lung cancer outcomes suggested in prior studies [42, 43].

Then, we conducted two sensitivity analyses to test the effects of PM_1_, PM_2.5_ and PM_10._ Firstly, we tested the sensitiveness of PM_1_ (PM_2.5_ or PM_10_) effect to the control of smoking and drinking factors. Behavior factors, including smoking prevalence, smoking strength (i.e. the number of cigarettes smoked per day) and drinking prevalence, are chosen mainly because of their indicated effects on lung cancer diseases in previous studies [47, 48]. Notably, second-hand smoking (instead of first-hand smoking) dominated the smoking-associated burden of lung cancer diseases for Chinese females [17]. Hence, we used total smoking prevalence (including the prevalence of the male and female) as the surrogate. Meanwhile, our smoking and drinking data accessible from the CHARLS are at city level and do not cover 436 registries (counties/districts) of this work. Hence, we attributed the same behavior characteristics (i.e. smoking and drinking) to registries belonging to the same city, leaving approximate 48% of the whole sample for the sensitivity analysis of smoking control. Secondly, we examined the robustness of PM_1_ (PM_2.5_ or PM_10_) effect to the adjustment of additional air pollutant (i.e. ozone).

Finally, the modifying role of urban-rural division on the effects of three particular matters on the incidence rate of female lung cancer was examined. We firstly stratified the whole dataset on the basis of urban-rural division. In line with the commonly used approach of urban and rural division in many prior studies [24, 27], we made use of counties and districts to delegate urban and rural areas, respectively. We then compared the effects of particular matter between urban and rural groups using Model 3. This is to investigate whether there is difference in particular matter effects. Secondly, we combined the stratified datasets and added the interaction between particular matter and urban-rural dummy variable in Model 3. This is to examine whether the difference in the effects of particular matter is significant. We did not comprise urban-rural dummy variable in Model 3, primarily resulting from its high collinearity with its interaction term (between particular matter and urban-rural dummy variable).

## Results

### Descriptive analysis

Table 1 shows the descriptive statistics of health outcome, PM_1_, PM_2.5_, PM_10_ and some socioeconomic covariates. As shown in Table 1, there was a great variation in incidence rate of female lung cancer among 436 Chinese cancer registries (counties/districts), with mean value and standard deviation by 22.42 per 10^5^ people and 8.85, respectively. Regarding three air pollutants, their mean values increased from 34.67 μg/m^3^ to 90.26 μg/m^3^ with the increase in particle sizes (Table 1). Considerable variations in PM_1_, PM_2.5_ and PM_10_ for 436 Chinese cancer registries were also observed, with the standard deviation by 11.14, 18.95 and 30.70, respectively (Table 1). With regards to socioeconomic covariates, there were also great variations observed for 436 registries between 2014 and 2016 (Table 1).

**Table 1:** Descriptive statistics of lung cancer disease, PM_1_, PM_2.5_, PM_10_ and some socioeconomic covariates

Variables	Mean	SD	Min	Max
Incidence rate of female lung cancer (per 10^5^ people)	22.42	8.85	0.00	81.84
PM_1_ (μg/m^3^)	34.67	11.14	8.56	71.67
PM_2.5_ (μg/m^3^)	45.80	18.95	2.40	94.64
PM_10_ (μg/m^3^)	90.26	30.70	34.29	207.84
Finance per capita (10^9^ RMB)	2.33	3.11	0.08	31.89
Average education years (10 years)	0.84	0.12	0.48	1.26
Construction workers% (10^−1^)	0.32%	2.18%	0.43%	31.45%
Manufacturing workers% (10^−1^)	7.98%	7.82%	0.25%	42.10%
Population (10^5^ people)	6.43	3.53	0.40	18.62

### Effects of PM_1_, PM_2.5_ and PM_10_

**Fig. 4:**
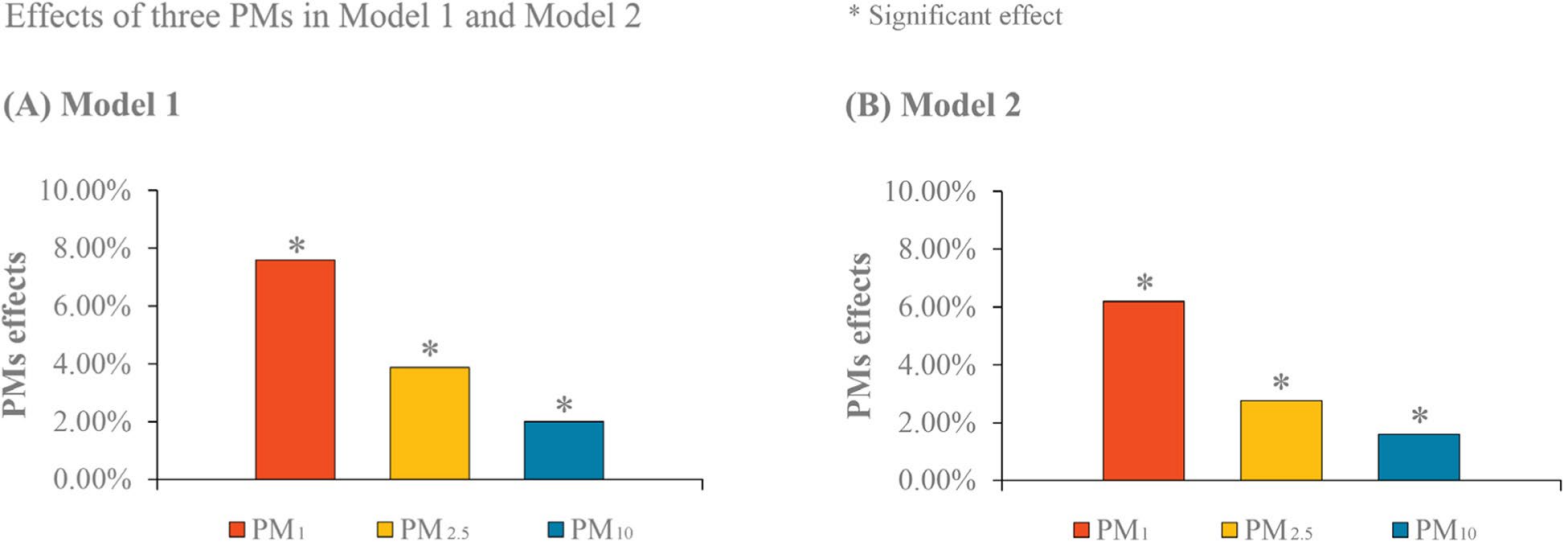
Effects of PM_1_, PM_2.5_ and PM_10_ in Model 1 and Model 2

The results of spatial associations between PM_1_, PM_2.5_ and PM_10_ and the incidence rate of female lung cancer are presented in Fig. 4 and Table 2. In General, the effects of the three particular matters increased, when there was an increase in particle sizes (PM_1_, PM_2.5_ and PM_10_). In the univariate model (Model 1, Fig. 4 (A)), when particular matter changed by 10 μg/m^3^, the change in the incidence rate of female lung cancer relative to its mean was 7.58% (95 CI%: 5.35, 9.77%) for PM_1_, which was larger than 3.88% (95 CI%: 2.58, 5.18%) for PM_2.5_ and 2.01% (95 CI%: 5.35, 9.77%) for PM_10_.

**Table 2:** Effects of PM_1_, PM_2.5_ and PM_10_ on the incidence rate of female lung cancer

Variables	PM_1_	PM_2.5_	PM_10_
	β (95% CI)	β (95% CI)	β (95% CI)
PMs	5.98% ***	3.75% ***	1.57% ***
	(3.40, 8.56%)	(2.33, 5.17%)	(0.73, 2.41%)
Log	0.43 ***	0.45 ***	0.47 ***
	(0.34, 0.52)	(0.36, 0.54)	(0.38, 0.55)
Year2015	−0.22	−1.42 **	−1.09
	(−1.69, 1.26)	(−2.75, −0.09)	(− 2.46, 0.28)
Year2016	1.32 **	0.34	0.49
	(−0.15, 2.80)	(−0.97, 1.65)	(− 0.88, 1.85)
Finance	0.04 ***	0.05 ***	0.05 ***
	(0.02, 0.07)	(0.03, 0.08)	(0.02, 0.07)
Education	−0.66 **	−0.60 **	− 0.54 **
	(−1.23, − 0.08)	(− 1.17, − 0.04)	(− 1.11, 0.03)
Construction	−0.03 **	− 0.03 **	−0.03 **
	(−0.06, 0.00)	(−0.06, − 0.01)	(−0.06, 0.00)
Manufacture	−0.02 ***	−0.03 ***	− 0.02 ***
	(−0.03, − 0.01)	(−0.04, − 0.02)	(−0.03, − 0.01)
Population	− 0.01	−0.03 ***	− 0.01
	(−0.03, 0.01)	(− 0.05, − 0.01)	(−0.03, 0.00)
Urban-rural	1.92 **	1.69 **	1.90 **
	(0.29, 3.55)	(0.07, 3.30)	(0.27, 3.54)

* for *p* < 0.1, ** for *p* < 0.05 and *** for *p* < 0.01. When PM_1_, PM_2.5_ and PM_10_ changed by 10 μg/m^3^, the change in the incidence rate relative to its mean = (10 × coefficient for PM_1_, PM_2.5_ and PM_10_)/mean incidence rate

In Model 2 controlling for location and time (Fig. 4 (B)), a similar pattern of results was observed, with the coefficients of 6.20% (95 CI%: 5.35, 9.77%) for PM_1_, 2.77% (95 CI%: 5.35, 9.77%) for PM_2.5_ and 1.60% (95 CI%: 5.35, 9.77%) for PM_10_. In Model 3 further adjusting for socioeconomic covariates, as shown in Table 2, the change in the incidence rate of female lung cancer relative to its mean was still largest for PM_1_, followed by PM_2.5_ and PM_10_, with the values of 5.98%, (95 CI%:3.40, 8.56%), 3.75% (95 CI%: 2.33, 5.17%) an 1.57% (95 CI%: 0.73, 2.41%), respectively, if there was a 10 μg/m^3^ change in the three particular matters.

### Sensitivity analysis

#### The control of smoking and drinking covariates

The finding that smaller particular matters have greater health effects was not sensitive to the control of smoking and drinking factors (Fig. S1). Specifically, when not controlling for smoking and drinking factors, as shown in Fig. S1 (A), the change in the incidence rate of female lung cancer relative to its mean was greater for PM_1_ than for PM_2.5_ and PM_10_. After the adjustment of three behavior covariates (Fig. S1(B)), a similar pattern of results was observed. If PM_1_, PM_2.5_ and PM_10_ changed by 10 μg/m^3^, then the change in the incidence rate of female lung cancer relative to its mean was 11.30% (95% CI: 7.37, 15.23%), 6.55% (95% CI: 4.55, 8.55%) and 4.12% (95% CI: 2.81, 5.42%), respectively (Fig. S1(B)). Meanwhile, as exhibited in Fig. S1(C) to Fig. S1(E), there were positive effects of certain smoking and drinking factors in each of the estimates of PM_1_, PM_2.5_ and PM_10_ effects.

#### The adjustment of additional air pollutants

Figure S2 exhibits the results of the sensitivity analyses of PM_1_, PM_2.5_ and PM_10_ effects to the adjustment of additional air pollutant. In general, the findings of finer particular matter having larger health effect were not sensitive to such adjustment. With the decrease in particle sizes, as presented in Fig. S2, the change in the incidence rate of female lung cancer relative to its mean increased from 1.13% (95% CI: 0.21, 2.05%) for PM_10_ to 5.18% (95% CI: 2.54, 7.82%) for PM_1_. We also observed the positive effect of ozone on the incidence rate of female lung cancer in each of the estimates of three particular matter effects (Fig. S2). Particularly, a 10 μg/m^3^ increase in ozone was positively associated with a 2.24% (95% CI: 1.68, 4.79%) increase in the incidence rate of female lung cancer relative to its mean (Fig. S2(B)).

### Modifying role of urban-rural division on the effects of PM_1_, PM_2.5_ and PM_10_

**Fig. 5:**
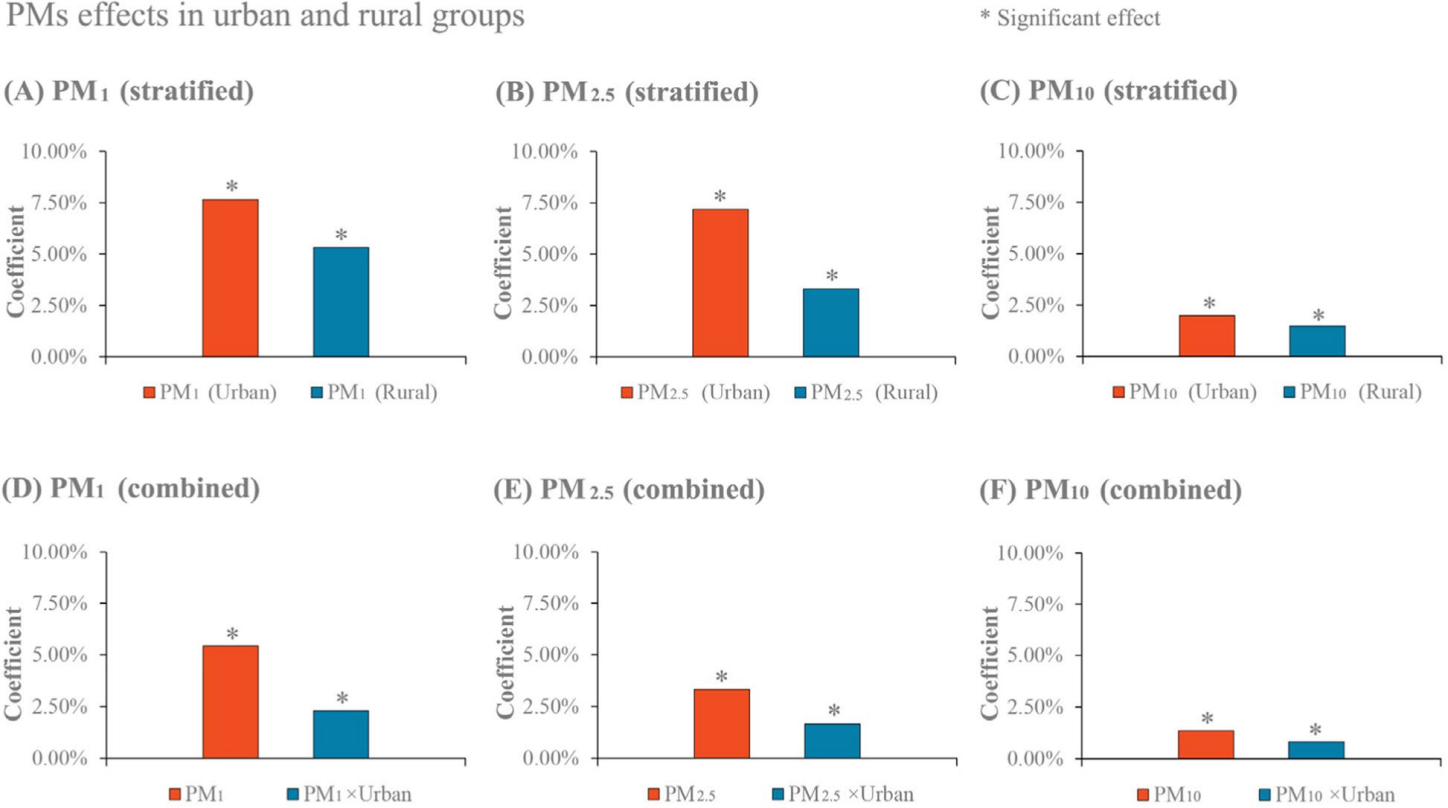
Modifying role of urban-rural division on the effects of PM_1_, PM_2.5_ and PM_10_

The results of urban-rural modification effects are shown in Fig. 5. In general, urban-rural division positively modified the effects of PM_1_, PM_2.5_ and PM_10_. In the stratified dataset, the effect of PM_1_ was positive in urban and rural groups with the effect greater for the former (Fig. 5(A)). In the combined dataset, as shown in Fig. 5(D), the change in the incidence rate of female lung cancer relative to its mean was greater by 2.29% (95% CI: 0.32, 4.27%) in urban than in rural group, if there was a 10 μg/m^3^ increase in PM_1_. A similar pattern of results was observed for PM_2.5_ and PM_10._ Particularly, PM_2.5_ and its interaction with urban-rural dummy variable were positively associated with the incidence rate of female lung cancer (Fig. 5(E)).

## Discussions

Many previous studies focus on PM_2.5_ and PM_10_ air pollutions in Chinese cities, while little attention has been placed on PM_1_. Currently, the strategies and standard already have been set for PM_2.5_ and PM_10_ in China, while the standard for PM_1_ is still missing. The nationwide or large-scale studies on health effects of PM_1_ benefit the establishment of PM_1_ standard and prevention measures all over China and even the world, while such studies are quite limited. Moreover, it remains unknown whether particular matters with smaller sizes are more harmful to human health, especially in China where particular matter air pollutions are still much more severe than western countries. As a response, this work as one of the earliest and largest nationwide studies in China, investigated the effects of PM_1_, PM_2.5_ and PM_10_ on the basis of data acquired from 436 counties/districts of China.

We observed the greatest effect of PM_1_, followed by PM_2.5_ and PM_10_. This is consistent with many previous Chinese studies [7, 10, 11]. In particular, a time-series study of eleven cities in Zhejiang Province of China suggested that the relative risk of all-cause mortality was higher for PM_1_ than for PM_2.5_ and PM_10_, when there was the same increase in each of the three particular matters [11]. Similarly, a short-term study conducted in two metropolitan cities of China (Guangzhou and Shenzhen) indicated that PM_1_ was more closely associated with emergency department visits from all cause than PM_2.5_ and PM_10_ [12]. The finding of more harmful effect of PM_1_ was further supported in other prior studies [13, 21, 49, 50]. Biologically, the high ratio of surface area to volume as well as the large percentage of toxic chemical composition in PM_1_ may be responsible for the greater effect of PM_1_. Our finding supports the argument that finer particular matters are more harmful than those with coarse particle sizes. Our finding also highlights that the regulation of particular matter air pollution in China should not only focus on PM_2.5_ and PM_10_, but also PM_1_. Standards, measures and strategies targeting the alleviation of PM_1_ should be highly prioritized because of the differences in chemical components and emission sources between PM_1_ and other particular matters in China, especially the control of emissions from automobile transport and industry which are usually identified as the main sources of PM_1_ air pollution.

We found that urban-rural division (rural group as the reference) positively modified the effect of PM_1_, PM_2.5_ and PM_10_. The finding of urban-rural modifying role is in line with those of some previous studies [43, 51, 52]. As suggested in a nationwide study of China [27], when there was a 10 μg/m^3^ increase in PM_2.5_, the relative risk of lung cancer incidence was 1.06 (95% CI: 1.04, 1.08) in urban areas, which is higher than the value of rural areas at 1.04 (95% CI: 1.00, 1.08). A Chinese study also reported that the effect of PM_10_ on the incidence of lung cancer was larger for urban inhabitants than for rural populations [53]. From a biological perspective, the difference in air pollution effects may be attributed to the varieties of material resources, biological factors and psychological stress among different socioeconomic groups. In China, the difference in particular matter effect between urban and rural groups may partly result from the difference in smoking behaviours (e.g. smoking prevalence and smoking strength), which has been discussed in our prior research [52]. The finding from the present study enhances the notion of the modifying role of socioeconomic factors on the effect of air pollution.

There are several limitations in the present study. Firstly, similar to most ecological research in relation to air pollution [54, 55], the well-recognized inherent errors in the estimates of particular matter exposures may be produced in our work. We operationalized PM_1_ (PM_2.5_ and PM_10_) exposure as the county (district)-aggregated mean concentrations of particular matter. Such operationalization did not take individual mobility into account and thus may produce exposure misclassification errors [56, 57]. Secondly, the estimates of PM_1_, PM_2.5_ and PM_10_ effects may be sensitive to the control of other potential socioeconomic and behavior covariates. In this work, our model construction is partly limited to the availability of socioeconomic and health data, with the number of variables by six and three, respectively. Hence, it may not be sufficient to control cofounders in relation to lung cancer diseases in the present study.

Thirdly, our work on the estimate of particular matter effect is an ecological study in nature, which may suffer from problems such as ecological fallacy [58]. However, ecological studies have their strengths of large sample size as well as big spatial coverage. Ecological studies, in combination with studies using individual-level data, may benefit an in-depth and more scientific understanding of the differential effects of PM_1_, PM_2.5_ and PM_10_ air pollution. Fourthly, the findings of PMs effects may be sensitive to the control of smoking and drinking factors, primarily resulted from the lack of smoking and drinking data for 436 registries. The sensitivity analysis, which derived city-level smoking and drinking data from the CHARLS, showed the robustness of PMs effects to the control of these behavior covariates. However, it is still not sufficient to well consider the effects of smoking and drinking factors. Attention should be paid if data these two covariates are available in the future.

## Conclusions

The association with the incidence rate of female lung cancer is stronger for PM_1_ than for PM_2.5_ and PM_10_ in China. There is positive modification effect of urban-rural division on the association between PM_1_ (PM_2.5_ and PM_10_) and the incidence rate of female lung cancer. On the one hand, the finding supports the argument that particular matters with finer particle sizes are more harmful to human health. On the other hand, the findings highlight that despite efforts on PM_2.5_ and PM_10_, the establishment of guidelines for PM_1_ is highly required and should be prioritized in China, especially the standard for PM_1_ as well as the strict control of emission sources of PM_1_ (e.g. automobile and industrial emissions).

## Supplementary Information

**Additional file 1.**

## Data Availability

The datasets generated and/or analysed during the current study are available in the “ChinaHighPM1 dataset” from https://weijing-rs.github.io/product.html, the “China Regional Estimates dataset (V4.CH.02)” from http://fizz.phys.dal.ca/~atmos/martin/?page_id=140, the “ChinaHighPM10 dataset” from https://weijing-rs.github.io/product.html and the “2015 China Health and Retirement Longitudinal Study (CHARLS) wave3” from http://charls.pku.edu.cn/en/page/data/2015-charls-wave4. These datasets are open access and freely available to all users.
