# Supplementary material for: Smaller particular matter, larger risk of female lung cancer incidence? Evidence from 436 Chinese counties
Source: BMC Public Health. 2022 Feb 18;22:344. doi: 10.1186/s12889-022-12622-1 (PMC8855598; doi:10.1186/s12889-022-12622-1)
Supplement: Supplementary file 1 — Additional file 1. [file 12889_2022_12622_MOESM1_ESM.docx]

**Supplementary Material**

**Smaller particular matter, larger risk of female lung cancer incidence? Evidence from 436 Chinese counties**

**1. Correlation and collinearity of PM_1_, PM_2.5_ and PM_10_**

Table S1 and S2 present the results of correlation and collinearity of PM_1_, PM_2.5_ and PM_10_. According to the results of correlation analysis (Table S1), PM_10_ is highly correlated with both PM_1_ and PM_2.5_, with Pearson Correlation Coefficient of 0.81 and 0.73, respectively.

On the basis of correlation analysis above, we further tested whether the findings of PM_1_ and PM_2.5_ were robust when considering the collinearity between the two pollutants. According to the results (Table S2), the effect of PM_1_ was still greater than that of PM_2.5_, with the effect of 3.26% (95% CI: 0.20%, 6.31%) and 2.78% (95% CI: 1.10%, 4.47%), respectively.

**Table S1. Correlation analysis between PM_1_, PM_2.5_ and PM_10_**

|  | PM_1_ | PM_2.5_ | PM_10_ |
| --- | --- | --- | --- |
| PM_1_ | 1 | 0.56 | 0.81 |
| PM_2.5_ | 0.56 | 1 | 0.73 |
| PM_10_ | 0.81 | 0.73 | 1 |

**Table S2. PMs effects to the consideration of collinearity between PMs in the model**

| Variables | β | (95% CI) |
| --- | --- | --- |
| PM_1_ | 3.26% ** | (0.20%, 6.31%) |
| PM_2.5_ | 2.78%*** | (1.10%, 4.47%) |
| Log | 0.43 *** | (0.34, 0.52) |
| Year2015 | -0.70 | (-2.19, 0.80) |
| Year2016 | 1.08 | (-0.40, 2.55) |
| Finance | 0.05 *** | (0.02, 0.07) |
| Education | -0.71 ** | (-1.28, -0.13) |
| Construction | -0.03 ** | (-0.06, 0.00) |
| Manufacture | -0.03 *** | (-0.04, -0.02) |
| Population | -0.02 ** | (-0.04, -0.01) |
| Urban-rural | 1.85 ** | (0.23, 3.46) |

* for *p* < 0.1, ** for *p* < 0.05 and *** for *p* < 0.01. When PM_1_, PM_2.5_ and PM_10_ changed by 10 μg/m^3^, the change in the incidence rate relative to its mean= (10×coefficient for PM_1_, PM_2.5_ and PM_10_)/mean incidence rate.


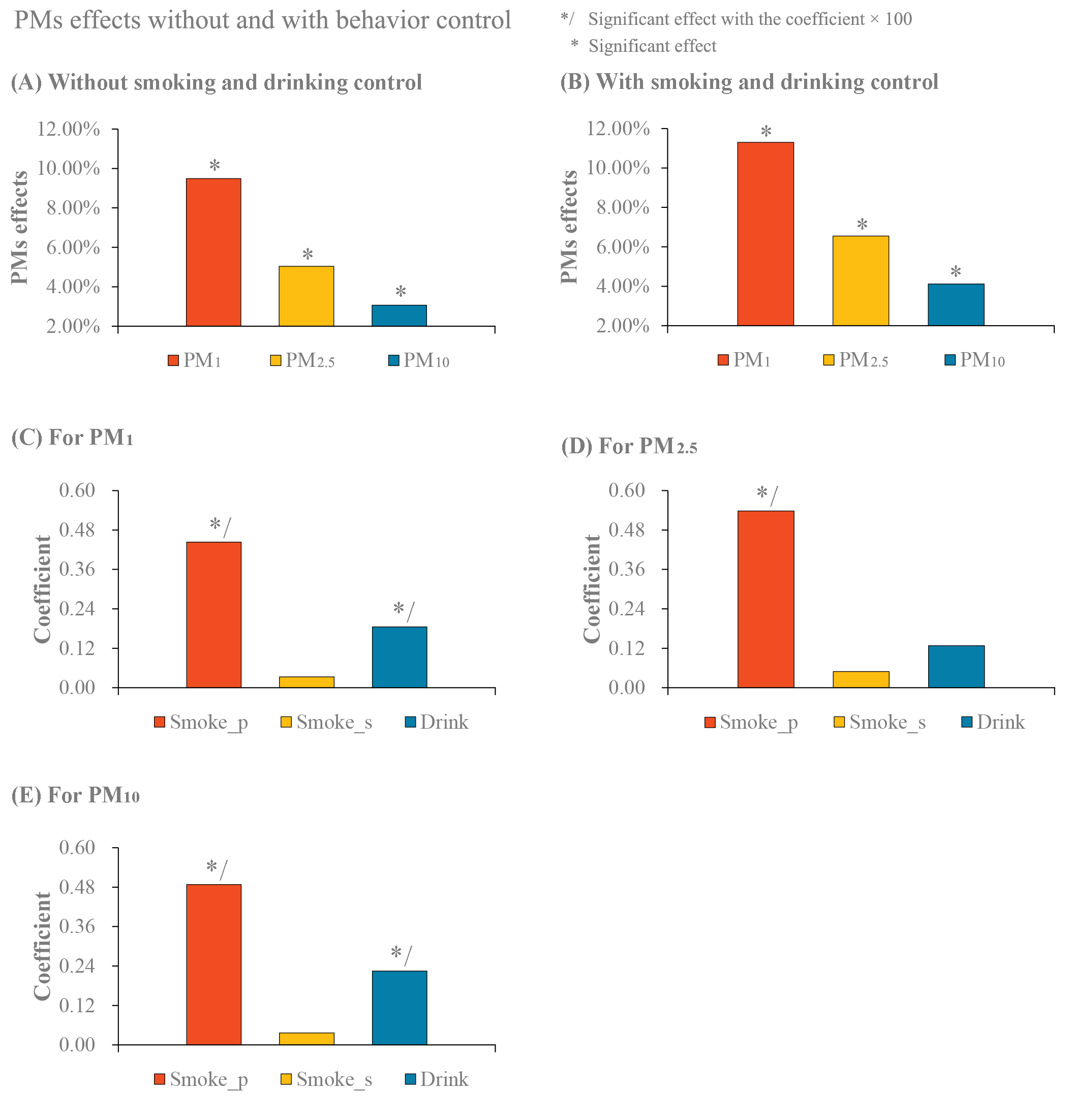


**Figure S1. Effects of PM_1_, PM_2.5_ and PM_10_ to the control of smoking and drinking covariates.**


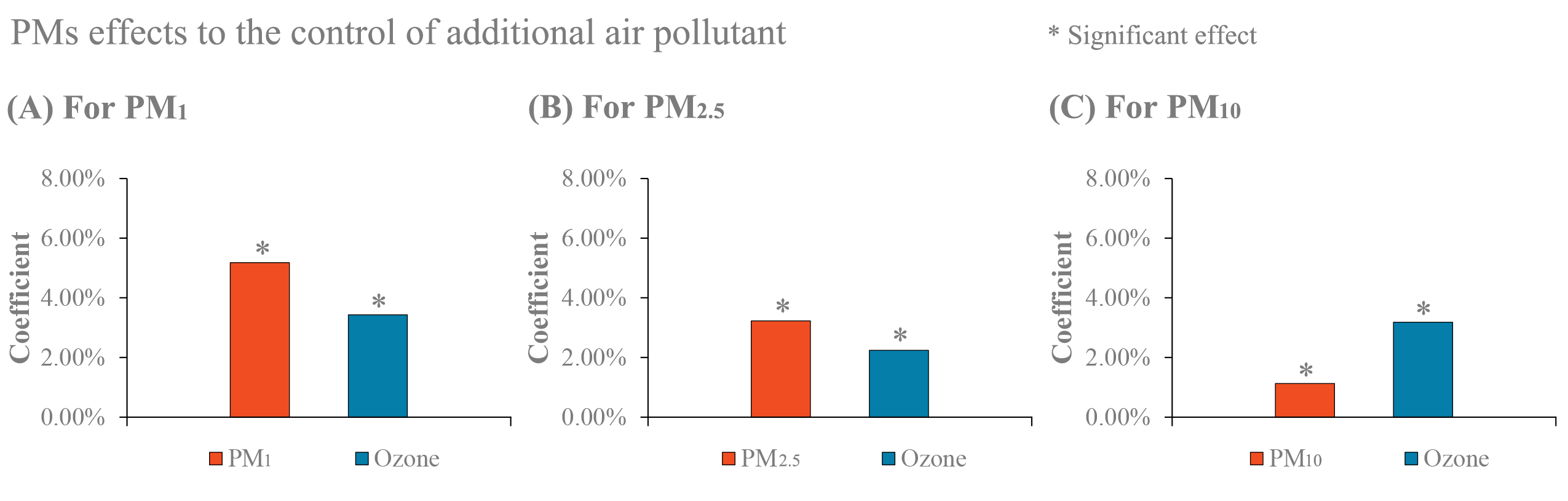


**Figure S2. PM_1_, PM_2.5_ and PM_10_ effects to the control of additional air pollution.**
